# RNA-seq analyses of blood-induced changes in gene expression in the mosquito vector species, *Aedes aegypti*

**DOI:** 10.1186/1471-2164-12-82

**Published:** 2011-01-28

**Authors:** Mariangela Bonizzoni, W Augustine Dunn, Corey L Campbell, Ken E Olson, Michelle T Dimon, Osvaldo Marinotti, Anthony A James

**Affiliations:** 1 Program in Public Health, University of California, Irvine, California, USA; 2 Department of Molecular Biology and Biochemistry, University of California, Irvine, California, USA; 3 Institute for Genomics and Bioinformatics, University of California, Irvine, California, USA; 4 Department of Biochemistry and Molecular Biology, Colorado State University, Ft Collins, Colorado, USA; 5 Department of Microbiology, Immunology and Pathology, Colorado State University Fort Collins, Colorado, USA; 6 Department of Biochemistry and Biophysics, University of California San Francisco, San Francisco, California, USA; 7 Biological and Medical Informatics Program, University of California San Francisco, San Francisco, California, USA; 8 Department of Microbiology and Molecular Genetics, University of California, California, Irvine, USA

## Abstract

**Background:**

Hematophagy is a common trait of insect vectors of disease. Extensive genome-wide transcriptional changes occur in mosquitoes after blood meals, and these are related to digestive and reproductive processes, among others. Studies of these changes are expected to reveal molecular targets for novel vector control and pathogen transmission-blocking strategies. The mosquito *Aedes aegypti *(Diptera, Culicidae), a vector of Dengue viruses, Yellow Fever Virus (YFV) and Chikungunya virus (CV), is the subject of this study to look at genome-wide changes in gene expression following a blood meal.

**Results:**

Transcriptional changes that follow a blood meal in *Ae. aegypti *females were explored using RNA-seq technology. Over 30% of more than 18,000 investigated transcripts accumulate differentially in mosquitoes at five hours after a blood meal when compared to those fed only on sugar. Forty transcripts accumulate only in blood-fed mosquitoes. The list of regulated transcripts correlates with an enhancement of digestive activity and a suppression of environmental stimuli perception and innate immunity. The alignment of more than 65 million high-quality short reads to the *Ae. aegypti *reference genome permitted the refinement of the current annotation of transcript boundaries, as well as the discovery of novel transcripts, exons and splicing variants. *Cis*-regulatory elements (CRE) and *cis*-regulatory modules (CRM) enriched significantly at the 5'end flanking sequences of blood meal-regulated genes were identified.

**Conclusions:**

This study provides the first global view of the changes in transcript accumulation elicited by a blood meal in *Ae. aegypti *females. This information permitted the identification of classes of potentially co-regulated genes and a description of biochemical and physiological events that occur immediately after blood feeding. The data presented here serve as a basis for novel vector control and pathogen transmission-blocking strategies including those in which the vectors are modified genetically to express anti-pathogen effector molecules.

## Background

Insect vector-borne pathogens cause some of the most widespread infectious diseases worldwide, including dengue fever, yellow fever, malaria, encephalitis, filariasis, leishmaniasis and trypanosomiasis [1,2]. The corresponding vectors are hematophagous insects that become infected by ingesting pathogens during blood feeding. Transmission of the pathogen to a subsequent vertebrate host occurs during the acquisition of another blood meal.

Hematophagy is a behavior exhibited by more than 14,000 species of insects [3-5], but genome-wide information regarding blood meal-regulated gene expression is available for only a few of these. Remarkable differences in the levels of accumulation of specific transcription products following a blood meal were reported in the malaria vector mosquito, *Anopheles gambiae *[6,7] and as many as 50% of all transcripts varied significantly during a gonotrophic cycle. Our study investigates blood meal-induced changes in transcript accumulation in the dengue vector mosquito, *Aedes aegypti*, that last shared a common ancestor with the Anophelines some 120-150 million years ago [8]. Elucidating transcriptional changes in mosquitoes following a blood meal can reveal novel molecular targets and strategies for control of vector populations and pathogen transmission. Alternative control strategies are required for dengue due to the continuous rise of cases worldwide [9,10], the current lack of an effective vaccine and the fact that vector control strategies aimed at reducing human contact with *Ae. aegypti*, the principal vector for all the four serotypes of Dengue viruses (DENV 1-4), have largely failed [11-13].

Previous studies analyzing the effects of blood meals on *Ae. aegypti *females were limited to the midgut [14], muscle mitochondria [15] or to specific gene sets [16,17]. Transcriptome sequencing, or RNA-seq, has emerged recently as a powerful tool to gain a holistic picture of the expression profile of an organism, tissue or cells [18,19]. Using next-generation sequencing technologies (Roche 454 GS FLX Genome Sequencer, Solexa/Illumina Genome Analyzer, ABI/SOLiD gene Sequencer and Helicos Genetic Analyses System), millions of cDNA reads of a length dependent on the platform chosen are generated and can be used either to create a *de novo *transcriptome assembly [20] or can be mapped to a reference genome to derive a genome-scale transcriptional map that consists of the structures of transcriptional units and their expression levels [21-23]. Sequencing-based methods provide absolute rather than relative gene expression measurements avoiding many inherent limitations of microarray technologies [24,25]. Additionally, RNA-seq data can be analyzed to assess differential-splicing activity, discover novel regions of transcription and locate precise transcription product boundaries [19,26].

We used the Illumina RNA-seq technology to compare the accumulation of transcription products in nonblood-fed female *Ae. aegypti *and mosquitoes at five hours post blood meal (PBM). This time point was chosen so that we may evaluate early genome-wide transcriptional responses to a blood meal. Results from our analyses assisted in refining the current annotation of the *Ae. aegypti *genome, improved our understanding of the biochemical pathways and biological processes elicited shortly after a blood meal and identified promoters and/or putative *cis*-regulatory elements correlated with changes in accumulation of specific gene products occurring as a consequence of ingestion of a blood meal.

## Results and Discussion

### Basic sequencing data

Four RNA-seq libraries were generated and sequenced from *Ae. aegypti *females of the Liverpool (LTV) strain. Two libraries were prepared from total RNA collected 3-5 day post eclosion from nonblood-fed females maintained with access to sugar (S) and the other two used RNA from females of the same age but at 5 hours after blood feeding (B). In total, 65,088,425 reads were generated and a close agreement between the technical replicates was confirmed by the Pearson correlation coefficients of 0.999 (S) and 0.995 (B) (Table 1, Additional file 1 Figure S1). Therefore, the data from parallel libraries were combined for further analyses.

**Table 1:** Mapping summary

**Condition**^**1**^	Replicate	**Total reads**^**2**^	**UMR**^**3**^	**No. transcripts**^**4**^	**PCC**^**5**^	**rRNA**^**6**^
B	R1	17400477	7172422	12,576 (69.63%)	0.995	1910
	R2	22240736	8366906	12,789 (70.81%)		
S	R1	10954762	3244060	11,914 (65.97%)	0.999	748
	R2	14492450	4332124	12,293 (68.06%)		

^1^Blood-fed (B) or sugar-fed (S).

^2^Total number of sequence reads.

^3^Uniquely Mapping Reads. Reads mapping to a unique position on the *Ae. aegypti *genome (assembly AaegL1).

^4^Number and percentage of the 18,061 *Ae. aegypti *annotated transcripts expressed as determined by the sequence reads (annotated transcripts derived from gene build AaegL1.2).

^5^Pearson Correlation Coefficient between replicates.

^6^Reads mapping uniquely to rRNA for the parallel B (R1+R2) and S (R1+R2) libraries.

### Differential transcript accumulation between nonblood-fed and blood-fed Ae. aegypti females

**Figure 1:**
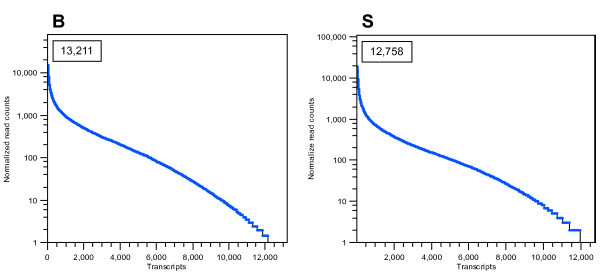
**Distribution of reads per transcript in RNA-seq libraries**. Blood-fed (B) and sugar-fed (S) transcripts are sorted from right to left in descending order of frequency of the total number of reads per transcript. The total numbers of transcripts detected for each experimental condition are shown in the insets.

RNA-seq analyses showed that ~ 70% of all annotated *Ae. aegypti *protein-encoding genes are expressed in both S and B mosquitoes (Figure 1). A total of 5969 transcripts were identified with differential accumulation between S and B mosquitoes, with 4160 and 1809 transcripts in greater or lesser abundance, respectively, following a blood meal (Additional file 2 Table S1). Quantitative reverse transcriptase PCR (qRT-PCR) on a random selection of thirteen genes showing differential accumulation levels confirmed both the direction and the magnitude of changes as shown by the Spearman rho correlation value of 0.975 (p < 0.001) and paired t-test value of 2.18 (p = 0.146) (Table 2).

**Table 2:** qRT-PCR validation of RNA-seq data on a random selection of thirteen genes

		RNAseq			qPCR		
		**Actual number of reads**^**1**^			**Normalized expression value**^**3**^		
Transcript	Function descriptors (Number of paralogous genes)	B (9,682,423)	S (4,280,814)	**Normalized fold changes**^**2**^	B (std)	S (std)	**Average fold changes**^**4**^
AAEL006138-RA	Vitellogenin-A1 (3)	118666	8	12.82	2.67 (0.44)	9.68^-4 ^(7.84^-4^)	11.43**
AAEL013284-RA	Serine-type endopeptidase AaLT (27)	22558	19	9.18	0.97 (0.61)	1.54^-2 ^(2.51^-2^)	5.98*
AAEL013707-RA	Trypsin-1 (28)	7612	32	6.86	1.28 (0.23)	6.96^-2 ^(3.50^-2^)	4.20**
AAEL001806-RA	Lipid binding (2)	171	1	6.38	0.19 (2.63^-2^)	3.39^-2 ^(1.04^-2^)	2.50**
AAEL014734-RA	Catalytic activity (0)	7770	802	2.24	3.08^-2 ^(2.63^-2^)	7.65^-3 ^(7.89^-3^)	2.01
AAEL011470-RA	Protein binding (0)	4551	615	1.85	5.91^-2 ^(2.39^-2^)	3.98^-2 ^(1.27^-2^)	0.57
AAEL013005-RA	Molecular function (0)	1833	253	1.82	1.15^-2 ^(3.55^-3^)	7.21^-3 ^(3.41^-3^)	0.67
AAEL002565-RA	Structural constituent of Cytoskeleton (16)	15421	21389	-1.51	6.52^-2 ^(1.66^-2^)	9.13^-2 ^(1.32^-2^)	-3.81*
AAEL008848-RA	Catalytic activity (0)	9414	11236	-1.29	0.30 (0.13)	0.62 (0.13)	-1.05*
AAEL012175-RA	Catalytic activity (4)	36942	41536	-1.21	0.35 (0.08)	1.42 (1.23)	-2.02
AAEL011871-RA	Electron Transporter (0)	11042	12116	-1.17	0.70 (0.40)	2.27 (0.42)	-1.70**
AAEL006425-RA	Serine-type endopeptidase (28)	1403	13914	-4.34	0.17 (4.62^-2^)	1.52 (0.39)	-3.16**
AAEL008701-RA	Iron Ion Binding (0)	51	689	-4.79	2.60^-3 ^(7.57^-4^)	0.196 (3.92^-2^)	-6.24**

^1^The total number of reads (in parentheses) identified in libraries prepared from blood- (B) and sugar-fed (S) mosquitoes.

^2^Normalized log_2 _fold changes.

^3^Normalized expression value as 2^ΔCT^, with the expression of the rp49 gene used to normalize values. Normalized expression values for blood-fed (B) and sugar-fed (S) mosquitoes were obtained as an average (±standard deviation) over four biological samples.

^4^Average fold changes as log_2 _(normalized expression value B/normalized expression value S). The significance of the difference in expression value between B and S mosquitoes was calculated using the t-test. *P < 0.05; **P < 0.01.

**Figure 2:**
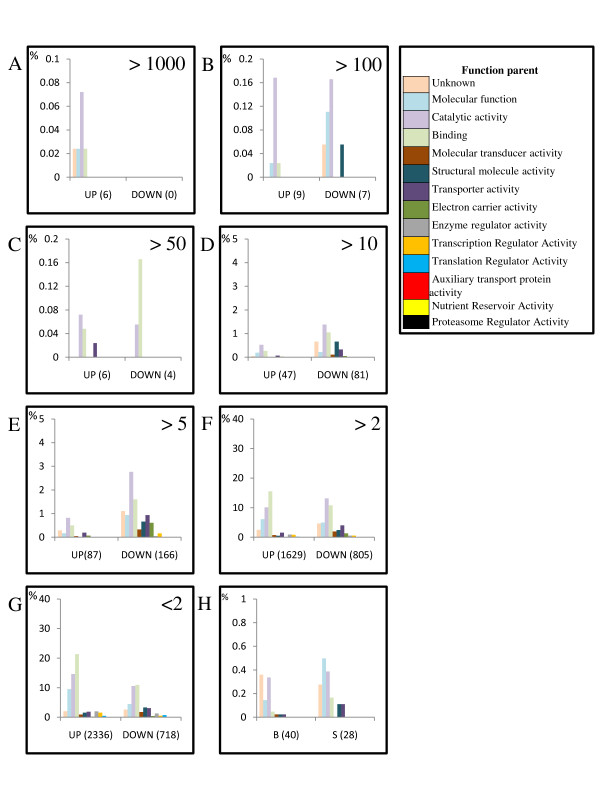
**Functional characterization of transcripts expressed differentially between blood- and sugar-fed *Aedes aegypti* mosquitoes**. Transcripts whose accumulation profiles were shown to be significantly increased (up) or decreased (down) were grouped according to the magnitude of fold-change. Each panel presents the proportion of transcripts assigned the function parent term indicated in the key [27] at different fold-change cut-offs (>1000, >100, >50, >10, >5, >2 and <2) shown in the upper-right corner (panel A-G). The data in each panel are non-cumulative, for example, Panel B shows those genes whose accumulation is less than 1000- but greater than 100-fold. Panel H shows the function parent of the transcripts found expressed significantly only in B or S mosquitoes as indicated.

Detailed examination of the 4160 transcripts showing increases in accumulation revealed that 21 are ≥50-fold more abundant in B mosquitoes, but that the majority (2336 transcripts) show less than a 2-fold increase. Forty transcripts are detected exclusively in B mosquitoes (Figure 2). Among the transcripts showing decreased accumulation following a blood meal, 971 were reduced between 2- and 5-fold in S when compared with B mosquitoes. Only 11 transcripts were decreased ≥50-fold, and 28 transcripts were represented exclusively in S mosquitoes.

The functions of proteins encoded by *Ae. aegypti *transcripts are predominantly theoretical and based on sequence similarities to those of other organisms. Acknowledging this limitation, functional parent attributions were assigned [27] for over 90% of the *Ae. aegypti *conceptual translation products allowing a description of the biochemical and physiological changes occurring following a blood meal (Figure 2). Blood feeding induced an accumulation of transcripts involved in lipid metabolism (acyl-CoA dehydrogenase, and aldehyde dehydrogenase), protein degradation (cathepsin, trypsins and serine proteases), ammonia/nitrogen metabolisms (glutamine synthetase and aspartate ammonia lyase) and egg maturation (vitellogenin). Based on the PFAM protein family database [28], the 21 transcripts whose abundance was increased ≥50 times in B versus S mosquitoes included those encoding two vitellogenins (AAEL010434-RA and AAEL006138-RA), 15 digestive enzymes, a member of the cytochrome P450 family (AAEL007812-RA), a sugar transporter (AAEL005533-RA) and one transcript (AAEL010435-RA) encoding an orthologue of the G12 gene of *An. gambiae *(AGAP006187). The G12 proteins in mosquitoes, thought to be secreted into the midgut lumen or maintained on the surface of microvilli, are encoded by transcripts that accumulate quickly in female midguts within one hour of blood feeding, reaching a maximum level of expression at about 12 hours PBM [29]. The same pattern of G12 expression is seen in *Ae. aegypti *females after feeding on blood infected with *Plasmodium gallinaceum *[30].

Transcript levels of genes whose products are involved in redox metabolism, such as dehydrogenases and members of the cytochrome P450 family, as well as those implicated in iron ion binding, increase between 5- and 2-fold, but several genes whose products are involved in similar physiology are decreased up to 10-fold. Furthermore, transcripts whose levels increased more than 5-fold are involved mainly in lipid and protein metabolism; levels of the majority of transcripts involved in trafficking/transport increased only slightly (less than 5-fold), if not decreased (Figure 2; Additional File 2, Supplemental Table 1). These observations are consistent with the conclusion that 5 hours PBM represents a time when *Ae. aegypti *females are beginning to respond actively to a blood meal through differential transcription. Additionally, the pattern of expression detected at the whole-body level 5 hours PBM reflects what is seen in *Ae. aegypti *midguts between 3 and 6 hours PBM [14], which is consistent with the conclusion that the blood meal is the event that signals the start of the metabolic activity. Transcripts involved in stimuli perception, such as those encoding odorant-binding proteins, were decreased, a finding that correlates with what is seen in *An. gambiae *females at 3 hours PBM [7]. Interestingly, transcripts associated with genes whose products are involved in transcription and translation also decreased at 5 hours PBM (Figure 2). The apparent contrast between the enhancement of digestive activity, which is centered in the midgut, and the decrease in transcripts linked to transcription and translation may reflect changes in transcript abundance occurring at the whole-body level.

### Transcripts found exclusively in blood-fed mosquitoes

Forty transcripts were found only in blood-fed mosquitoes, with the highest read-counts reaching ~1000/transcript, after normalizing for different library sizes (Additional File 2 Supplemental Table 1). Functional parent attribution for these transcripts is consistent with a role in digestion and in the progression of the gonotrophic cycle. Specifically, two transcripts, Aa5G1 (AAE013712-RA) and AaSPVI (AAE010196-RA), correspond to the midgut serine proteases shown previously to be elicited by a blood meal in the midgut of *Ae. aegypti *females [17]. Seven other transcripts encode enzymes (i.e. decarboxylase, cathepsin b and trypsins), and two are implicated in trafficking. Transcripts AAE014815-RA and AAE005950-RB correspond to the vacuolar protein sorting 13B from yeast and the chloride channel protein 2, respectively. Ten transcripts are paralogous to the G12 gene of *An. gambiae *and share the Insect Allergen Repeat motif. This motif is hypothesized to be a novel, insect-specific detoxifying domain implicated in the co-evolution of herbivorous insects and their plant hosts and also has been linked to nitrile-specific detoxification [31]. Transcripts AAEL006126-RB and AAEL008921-RC are predicted orthologues of the *Culex quinquefasciatus vitellogenin-A1 *gene and the *Drosophila melanogaster spaghetti squash *(*sqh*) gene, respectively. The *sqh *gene product encodes the regulatory light-chain of non-muscle myosin II, which is required for cytoplasmic transport in nurse cells during oogenesis and also has been implicated in germline RNA interference (RNAi) processes [32].

### Transcripts found exclusively in sugar-fed mosquitoes

Twenty-eight transcripts were found to accumulate significantly only in sugar-fed mosquitoes. Parent attribution is consistent with roles in basal metabolism and stimuli perception. In particular, six of the 28 transcripts encode proteins with catalytic activity (peptidase and protease), three belong to the cytochrome P450 protein family (AAEL014684-RA, AAEL013555-RA, AAEL000320-RA), and five (AAEL000350-RA, AAEL003311-RA, AAEL000318-RA, AAEL006108-RA, AAEL009597-RA) are conserved hypothetical proteins that share the Insect pheromone/odorant binding protein (PhBP) domain [33]. Two of the 28 correspond to putative cuticle proteins (AAEL000879-RA, AAEL013520-RA), and one transcript (AAEL013434-RA) encodes a protein similar to the product of *Spätzle *1A, which is required for the Toll-dependent antimicrobial response in both adult and larval vinegar flies [34,35]. Two transcripts (AAEL8931-RA and AAEL10995-RA) encode proteins with predicted transporter activity. The functions of the proteins encoded by the remaining nine transcripts are unknown.

### Transcripts related to pathogen interaction

Blood feeding is the primary port of entry into mosquitoes for viral, protozoan and metazoan pathogens that cause diseases in vertebrates. While blood is a source of nutritive resources for mosquitoes, it also is potentially harmful to them, and a balance between these factors determines their fitness [36]. Two mechanistically different innate immune defense mechanisms have been described in *Ae. aegypti*: one relies on gene expression control and degradation of mRNA through the small RNA regulatory pathways (SRRPs) [37,38] and the other induces the production of antimicrobial peptides and/or promotes phagocytosis, encapsulation and melanization of pathogens through the Toll, Imd and JAK-STAT signaling pathways [39-41]. The activities of the genes in these pathways have been analyzed in *Ae. aegypti *challenged by injection with various pathogens including bacteria [39,42], the filarial worm *Brugia malayi *[43], Sindbis and dengue viruses [37,40,44-47]. Transcriptional activation of innate immunity genes occurs within minutes after infection and the response lacks immunologic memory [39]. Additionally, it has been hypothesized that the natural bacterial flora in mosquitoes maintains a basal level of immune response [44,48] and that immunity processes share bio-products, such as reactive oxygen species (ROS), with digestion [49]. As a consequence, analyzing the basal expression of immunity genes shortly after a blood meal could help identify elements that govern vector competence and clarify the level of synergy among immunity and digestive processes. Early transcriptional responses to a blood meal are relevant particularly with respect to dengue infection as viruses can be internalized within 5-7 minutes of contact between the virions and the mosquito midgut epithelial cells [40], and viral replication is evident in the midgut two days post infection [50].

**Figure 3:**
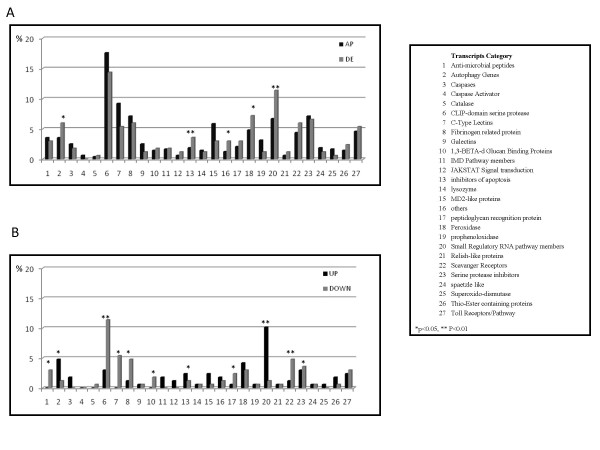
**Immune-related transcripts differentially accumulated between blood- and sugar-fed *Aedes aegypti* females**. Transcripts were classified based on categories established by comparative genomic analyses *in silico *[46,51,52]. (A) Percentage distributions of all transcripts (n = 477) with a putative or characterized Anti-Pathogen (AP) function and only those AP that are differentially-expressed (DE) (n = 167). (B) Percentage distribution with respect to the total of 167 DE immunity transcripts that increase (up) or decrease (down) in abundance at 5 hours PBM. Significant enrichments in number of transcripts per class are indicated by the asterisks (* p < 0.05; ** P < 0.001).

Among the 477 transcripts identified by comparative genomic analyses *in silico *and manual annotation that have established or putative associations with defense mechanisms [27,33,37,38,40,44,46,47,51,52] (Additional file 3 Table S2), 167 were expressed differentially with 88 and 79 showing lesser and greater accumulation in blood-fed mosquitoes, respectively (Figure 3). Several classes of genes, including those encoding receptors and effectors of the immunity cascade (scavenger receptors, CLIP-domain serine proteases, peptidoglycan recognition proteins, fibrinogen-related protein, C-type lectins, 1,3-β-d glucan binding protein and anti-microbial peptides) [46,51,52], were represented highly among those that showed decreased transcript accumulation following the blood meal (Figure 3). Fold-changes ranged between 1.09 (AAEL008738-RA) and 24.61 (AAEL011375-RA [CLIPD11]), with the majority (52 transcripts) decreasing more than 2-fold. One transcript (Spätzle 1A [AAEL013434-RA]) was found exclusively in sugar fed mosquitoes. Fourteen transcripts decreased >5-fold, including two members of the CLIP-domain serine protease (CLIPB35 [AAEL000037-RA] and the previously-mentioned CLIPD11) and three C-Type lectins (CTLMA13 [AAEL011621-RA], CTL18 [AAEL005482-RA] and CTMLA12 [AAEL011455-RA]).

Fold-changes for the 79 transcripts showing increased accumulation vary between 1.16 and 29.32, the former corresponding to transcript AAEL008073-RA, a SRRP member, and the latter to transcript AAEL015136-RA, belonging to the MD2-like protein (MLs) group. MD2-like genes encode secreted proteins containing a lipid recognition domain that acts as intermediate in the immune response. The observed expansion of the mosquito MD-2 gene family may indicate a specialized function of their products in the defense against pathogens ingested with blood meals [51]. Three other MD2-like transcripts (AAEL003325-RA; AAEL004120-RA; AAEL009531-RA) increase in abundance at 5 hours PBM, although not more than 2.3 fold. In addition to AAEL015136-RA, only two other transcripts (AAEL000859-RA and AAEL003255-RA), not classified in any of the canonical immunity gene categories [46,51,52], accumulate more than 5-fold (Additional file 3 Table 2). The majority of transcripts (52 out of 79) accumulated less than 2-fold higher in blood- versus sugar-fed mosquitoes. The negative regulators of the Toll and IMD pathways, Cactus (AAEL000709-RA) and Caspar (AAEL0014734-RA), were 1.52-and 4.72-fold, respectively, more abundant.

A number of genes involved in autophagy, SRRP members and inhibitors of apoptosis had transcripts whose accumulation increased significantly following a blood meal (Figure 3B; Additional file 2 supplemental Table 1). The maximum increase observed, 3.10 fold, was detected for the inhibitor of apoptosis IAP2 (AAEL006633-RA). Autophagy is a tightly-regulated catabolic process whereby cells degrade intracellular components via the lysosomal machinery and it plays an important role in homeostasis maintenance, cell development, growth and immunity [46,52,53]. The increase in accumulation of autophagy genes and of members of the inhibitors of apoptosis is not surprising considering the time-point, 5 h PBM, sample here. Among the 17 SRRP members showing increased transcript accumulation, four, Dicer 2 (AAEL006794-RA), TSN (AAEL000293-RA), Dicer1 (AAEL001612-RA) and PIWI4 (AAEL007698-RA), were at least 2-fold more abundant following a blood meal. Dicer2 and TSN are essential components of the RNA interference (RNAi) effector multi-component RNA-induced Silencing Complex (RISC) [38,47], and Dicer1 has been shown to control gene expression of 'housekeeping' genes [38]. PIWI4 is a member of the PIWI small RNA (piRNA) pathway proposed to be involved in anti-viral defense [38].

### Cis-regulatory element discovery

Tightly-regulated and blood meal-induced expression profiles are of particular interest for designing transgenic mosquito-based control strategies to reduce transmission of dengue fever. *Cis *regulatory sequences derived from blood meal-induced/up-regulated mosquito genes allow potentiating swift induction and effective levels of transcription of an associated effector gene, while likely inflicting the least fitness cost [54,55]. We interpret the different levels of mRNA accumulation seen in this study to reflect changes in transcriptional activity of the corresponding genes, although it is possible that some levels may vary as a function of changing transcript stability or rates of turnover. With this in mind, we used SCOPE [56] to predict putative CREs that may provide the basis for rational identification and selection of new candidate promoter regions and for modification of the transcriptional profiles of current transgene constructs. We examined the 2000 base pairs (bp) flanking the 5'-boundaries of the 40 transcripts that were undetected in libraries from sugar-fed mosquitoes but detected at significant levels in the RNA-seq libraries from blood-fed mosquitoes and identified a redundant list of 22 motifs that are enriched significantly in these sequences (Additional File 4 Figure 2). A possible *cis*-regulatory module (CRM) constructed with the discovered CREs is represented by the motif consensus sequences, cnatcnkcwgtt, gyactyvar, and tgakamga, and is associated with *Ae. aegypti *paralogues of the G12 gene of *An. gambiae *(AGAP006187) (Additional File 4 Figure 2). *Aedes aegypti *has 17 G12 genes, many more relative to other insects, which have 4.5 on average (according to OrthoDB; group EOG95TCTG) [57]. The transcripts of nine of the G12 paralogues are present in this co-regulated gene set (representing ~25% of the 40).

Another putative CRM contains the consensus sequence tgakamga, cnatcnkcwgtt, asttrccc and aarcttbd (Additional File 4 Figure 2). This CRM groups with the cathepsin b genes, AAEL015312-RA and AAEL007585-RA. Verification of these CRMs will require empirical testing, however, the top 10 matches for tgakamga, which is present in both putative CRMs, align well to members of the mosquito-conserved GATA motifs correlated to transcriptional responses to blood feeding in *An. gambiae *[58].

### RNA-seq identifies annotation corrections

RNA-seq also provides an opportunity to examine and improve the current annotation of the *Ae. aegypti *genome and examine the level of transcriptome plasticity in terms of alternative splicing. We used HMMSplicer [58] to compare junctions revealed by our data to the annotation provided by Vectorbase and Ensembl [33,60]. HMMSplicer predicted 32,501 junctions supported by at least two RNA-seq reads using the combined data from sugar and blood-fed samples. Of these, 24,100 (74%) matched junctions present in the AaegL1.2 gene-build provided by VectorBase, leaving 8,401 predicted novel high-scoring splice sites supported by multiple RNA-seq reads [61]. A total of 4500 (~54%) of these occur within annotated gene boundaries and may represent un-annotated alternatively-spliced transcripts. To estimate how many of the remaining splice junctions might be truly novel, we mapped them to increasingly larger DNA fragments flanking the currently-annotated genes (Table 3). A total of 2687 (~33%) junctions mapped within 32,000 bp of the 5'- or 3'-ends of annotated gene boundaries. Of these, 1439 mapped within 4000 bp, consistent with the interpretation that they may represent alternatively-spliced transcripts of the previously-identified genes. Those mapping beyond 4000 bp could be alternate junctions of the known genes, represent un-annotated transcription products or be artifacts.

**Table 3:** Predicted novel junctions within varying distances from annotated transcripts^1^

Distance from annotation (±bp)	Predicted junctions
0-1000	1170
1001-2000	120
2001-4000	149
4001-8000	245
8001-16,000	486
16,001-32,000	517
>32000	854

**Total**	**3541**

^1^Extending to either the 5'- or 3'-end of the annotated gene (Gene build AaegL1.2).

**Figure 4:**
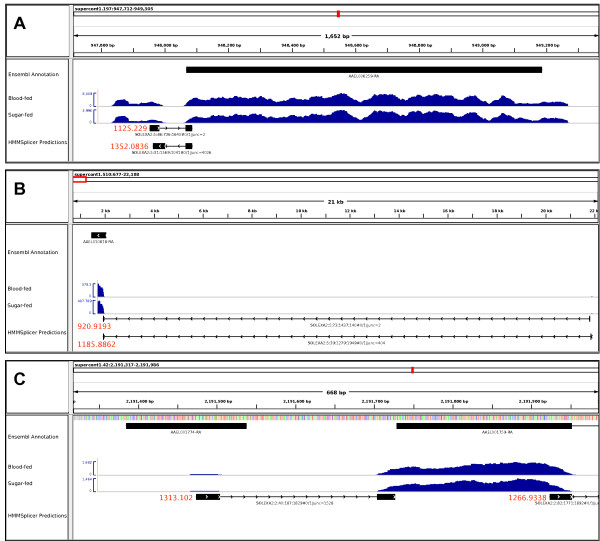
**Examples of amendments to the Ae. aegypti annotation supported by HMMSplicer results**. Black bars in the top tracks represent the current gene annotations. Blue histograms in the second track represent the non-normalized coverage of RNA-seq reads at each position. The range of the histogram values shown in each view is depicted on the labeled y-axis of each RNA-seq track. Black boxes in the lower track represent splice-site predictions based on the RNA-seq reads using HMMSplicer determined in this study. Each function has a unique identifier listed below and its HMMSplicer score is listed in red. If multiple reads support a single junction, "junc = x" lists the number of supporting reads. This information provides evidence to link two islands of transcription as a single transcription event, therefore, exons of a common mRNA. All predicted junctions shown here also are supported by EST alignments. Genes are (A) AAEL006259; (B) AAEL010818; and (C) AAEL001774 and AAEL001759.

An accurate gene annotation, especially with respect to the transcription start site (TSS), is paramount for the accurate discovery of CREs because prediction tools must make the assumption that the sequences included are true regulatory regions, and their performance suffers when this is false. For the CRE predictions described in the previous section, 36 of the 40 transcript start sites were in close agreement to the Ensembl annotation [60]. Figure 4 highlights three determined amendments to the current annotation, all supported by EST data. Figure 4A and 4B supports the conclusion that the current annotation has missed the putative first exons that extend the 5'-UTRs of some genes (AAEL006259, AAEL010818) and provides additional information for predicting accurate transcriptional start sites (TSS). In the case of AAEL010818, the TSS determined by RNA-seq data is 20 kb to the 5'-end of the annotated start site, far outside the distances commonly searched for CREs (Figure 4B). In some cases, as was seen for AAEL001774, the first exon was annotated but included as a separate gene model, which also contains the likely 5'-UTR of AAEL001759 (Figure 4C). AAEL001774 encodes a protein comprising 50 amino acids with no known functional domains aside from a predicted signal peptide that makes up 66% of its length.

## Conclusions

We provide a detailed examination of the changes in transcripts accumulation occurring at the whole-body level of *Ae. aegypti *females 5 hours PBM. The observed changes are consistent with the beginning of an intense physiological response to a blood meal. The majority of immunity-related transcripts tended to accumulate at lower levels in blood fed mosquitoes. This finding supports the hypothesis that there may be a gap in immunity following a blood meal. Reduced expression of immune genes in blood fed mosquitoes could favor the establishment of infections, especially considering that pathogens such as dengue viruses infect the midgut epithelial cells within minutes after the contact [50]. However, changes in transcript abundance observed at the whole-body level may mask changes in accumulation occurring primarily in the midgut. Different levels of activation of immunity genes after a blood feeding may be one of the factors contributing to the variability in vector competence for dengue viruses observed in different geographic populations of *Ae. aegypti *[62,63]. The quantity and quality of data generated by RNA-seq technology makes this an ideal approach for comparative analyses of the transcriptome of *Ae. aegypti *strains with different vector competence and vectorial capacity.

Our analyses of the expression profiles of S and B mosquitoes allowed the identification of co-regulated genes and putative *cis*-regulatory elements and modules from the *Ae. aegypti *genome. Further knowledge of the mechanisms involved in regulation of gene expression in vector species is critical to the development of control strategies whereby the vector is modified genetically to express anti-pathogen effector molecules in tissue-specific and time-regulated manners [64]. Promoter and other *cis*-acting regulatory DNA fragments are needed to regulate restricted expression of selected anti-pathogen effector molecules. Moreover, we described several examples of how the RNA-seq data generated can help improve the current annotation of the *Ae. aegypti *genome.

## Methods

### Mosquito strains and rearing

The *Ae. aegypti *Liverpool strain (LTV) used in this study originated from West Africa where it was selected for susceptibility to the filarial worm parasite, *Brugia malayi *[65], and has been maintained at the Liverpool School of Tropical Medicine since 1936. DNA from mosquitoes of this strain, derived after twelve consecutive generation of single-pair inbreeding, was used to generate the currently available *Ae. aegypti *genome sequence [66]. Mosquitoes were maintained at 28°C, 70-80% relative humidity, with 12-12 h light-dark photoperiod at Colorado State University (Fort Collins, Colorado). Larvae were fed on a finely-ground fish food (Tetramin, Tetra Werke, Germany). Males and females were kept together in a cage with unlimited access to water and sugar (raisin) until blood feeding. Mosquitoes aged 3-5 days after eclosion were allowed to feed on immobilized mice. The study was carried out in strict accordance with the recommendations in the Guide for the Care and Use of Laboratory Animals of the National Institutes of Health. Female mosquitoes were flash-frozen in dry ice and promptly stored (-80°C) five hours after blood feeding and shipped to the University of California, Irvine for RNA extraction.

### RNA extraction and Illumina library preparation

Total RNA was extracted with TRIZOL (Invitrogen) from pools of three females (3-5 days old) either exclusively kept on a sugar diet (S) or five hours after blood feeding (B). After checking for the quality of RNA with an Agilent 2100 bioanalyzer, two samples of S and B were pooled to reach the 20 micrograms necessary for the preparation of two single-read Illumina libraries [67]. Illumina libraries were prepared and run for 40 cycles by the Expression Analysis Core at the UC Davis Genome Center [68]. Libraries were run at a concentration of 4-5 pM.

### Processing of Illumina sequencing data

Sequencing data were retrieved from the UC Davis Genome Center through r-sync. Sequencing data have been deposited at the Short Read Archive (NCBI) under accession number GSE24872. Data from the two technical replicates were combined to gain sequencing depth after having verified the technical reproducibility of the two libraries generated for each condition (B and S). Bowtie [69] was used to align the Illumina reads against the *Ae. aegypti *genome (version AaegL1) [33], allowing a maximum of two mismatches and with the -m option, which returns only reads with a single best match in the genome. Reads mapping to ribosomal RNA genes were filtered out from the Bowtie output using a custom Python script. The percentage of covered transcriptome was determined using BEDTools [70]. Differential expression between conditions was assessed by the likelihood ratio test as implemented in the program DEGseq [71], after accounting for the different total gene counts of each library, at a p value of 0.001 and with a false discovery rate (FDR) of 0.1% [72]. Transcript description was based on the *Ae. aegypti *protein database AegyXcel [27].

### Real-time quantitative RT-PCR validation of RNA-seq data

A total of 13 genes identified by RNA-seq to be expressed differentially between S and B mosquitoes were chosen for real-time quantitative PCR analysis (Additional File 5 Table S3). Total RNA was extracted by TRIZOL (Invitrogen) from a pool of eight females kept exclusively on a sugar diet or a similar pool collected five hours after blood feeding. Following DNAse I (Invitrogen) treatment, a total of 10 μg of RNA were used for cDNA synthesis with superscript III (Invitrogen) and random primers. Real-time quantitative PCR reactions of 20 μl were performed in triplicate with SYBR Green Supermix (Biorad) and 0.3 μM of each primer on three sequential five-fold dilutions each of the original cDNA. Real-time quantitative PCR reactions were run on an iQ3 system (Biorad). No primer dimer was detected when inspecting the melting curves and primer pairs were chosen that displayed greater than 90% amplification efficiency, in all cases except AAEL002565, where efficiency was 89.313 ± 5.384 (Additional File 5 Table S3). Fold-changes in gene expression between S and B mosquitoes were derived by the comparative C_T _method [73], using the constitutive gene rp49 (GenBank Acc. No.:AY539746; AAEL003396) as the reference and four samples each for S and B mosquitoes. Correlation between the expression values detected by RNA-seq and qRT-PCR for the 13 genes tested was estimated by calculating Spearman's Rho correlation in the JMP501 statistical software (SAS Institute INC., Cary, NC). The paired t-test in Excel was used to compare the expression values for each transcript in the two methods. The significance of the qRT-PCR-based difference in expression values between B and S mosquitoes based on four samples each for B and S were calculated using a standard t-test.

### Splice-site predictions

The program HMMsplicer [59] followed by custom Python scripts was used to assess transcriptome plasticity. Initial HMMsplicer runs were performed separately for sugar-fed and blood-fed samples using all RNA-seq reads that passed Illumina's quality filtering, regardless of whether they aligned to the genome. Junctions were predicted initially for single reads and then combined with perfectly matching junctions and junctions within 3 bp of each other. The combined junction inherits the location of the highest scoring junction and the combined score is adjusted appropriately. Only junctions predicting canonical splice sites after this combination were retained. Predictions for sugar-fed and blood-fed samples were combined and scores adjusted similar to above to improve the predictive power, but perfectly matching junctions were required for junctions to be combined. Finally, only junctions with more than one supporting RNA-seq read and an HMMsplicer score of 600 or greater were considered here.

### Motif discovery

SCOPE [56] uses an ensemble method to combine the results of three specialized motif finders that separately concentrate on non-degenerate motifs, degenerate motifs and motifs that contain two separate "half-sites". It generates significance scores by combining overrepresentation, positional bias and the proportion of the co-regulated promoters to contain at least one instance of the motif. It is resistant to the common problem of extraneous or "non-informative" promoter regions included in the co-regulated set. SCOPE was run using the 2000 bp upstream of the start codon for each transcript with SCOPE's OccurrenceKSScorer to generate the significance values.

## Authors' contributions

MB performed the experiments, analyzed the data and wrote the manuscript. WAD wrote custom python codes for data analyses, performed the bio-informatic analyses of the data and wrote the manuscript. CC provided staged insects for mRNA extractions. KEO reviewed the manuscript and provided mosquito resources. MTD performed the HMMSplicer analyses. OM conceived the study, analyzed the data and reviewed the manuscript. AAJ conceived the study and wrote the manuscript.

## Supplementary Material

Additional file 1 **Comparison of normalized transcript abundance between replicate libraries with respective Pearson correlations**. (B) Blood-fed. (S) Sugar-fed. Axes values are in reads transcript^-1 ^library^-1^. B_a_: blood-fed replicate library A. B_b_: blood-fed replicate library B. S_a_: sugar-fed replicate library A. S_b_: sugar-fed replicate library B. The Pearson statistics and equation for the best-fit line are shown in the inset. Click here for file

Additional file 2 **List of all the transcripts identified from RNA-seq libraries from blood- and sugar-fed *Aedes aegypti *females**. The number of reads per transcript and the fold-changes in gene expression between blood- and sugar-fed samples also are included. Sheets 2 and 3 list the transcripts found at significant levels only in blood- and sugar-fed mosquitoes, respectively. In sheets 2 and 3, a column with the transcript description as derived from Ensembl Metazoa [60] and three columns with values corresponding to "function parent", "best match to SWISSP database" and "best match to PFAM database" as derived from AegyXcel [27] are included. Click here for file

Additional file 3 **List of the transcripts related to pathogen interaction or with putative defense mechanisms, as identified by a comparative genomic analyses *in silico ***[27,37,38,40-47,51,52]. Sheet 2 and 3 list the Anti-Pathogen (AP) transcripts with increased and decreased accumulation in blood fed mosquitoes, respectively. Click here for file

Additional File 4 **Motif map of putative CREs discovered by SCOPE using transcripts detected significantly only in blood fed female *Ae. aegypti***. Locations of representative SCOPE-derived CRE motifs in the 2000 bp upstream of the annotated translational start site in the 40 transcripts detected significantly only in B. Transcript names on the left are ordered from most (top) to least (bottom) abundant. Click here for file

Additional file 5 **List of primers used for real-time RT-PCR validation of RNA-seq based data**. Click here for file
