# Supplementary figures and images for: RNA-seq analyses of blood-induced changes in gene expression in the mosquito vector species, Aedes aegypti
Source: BMC Genomics. 2011 Jan 28;12:82. doi: 10.1186/1471-2164-12-82 (PMC3042412; doi:10.1186/1471-2164-12-82)

**B**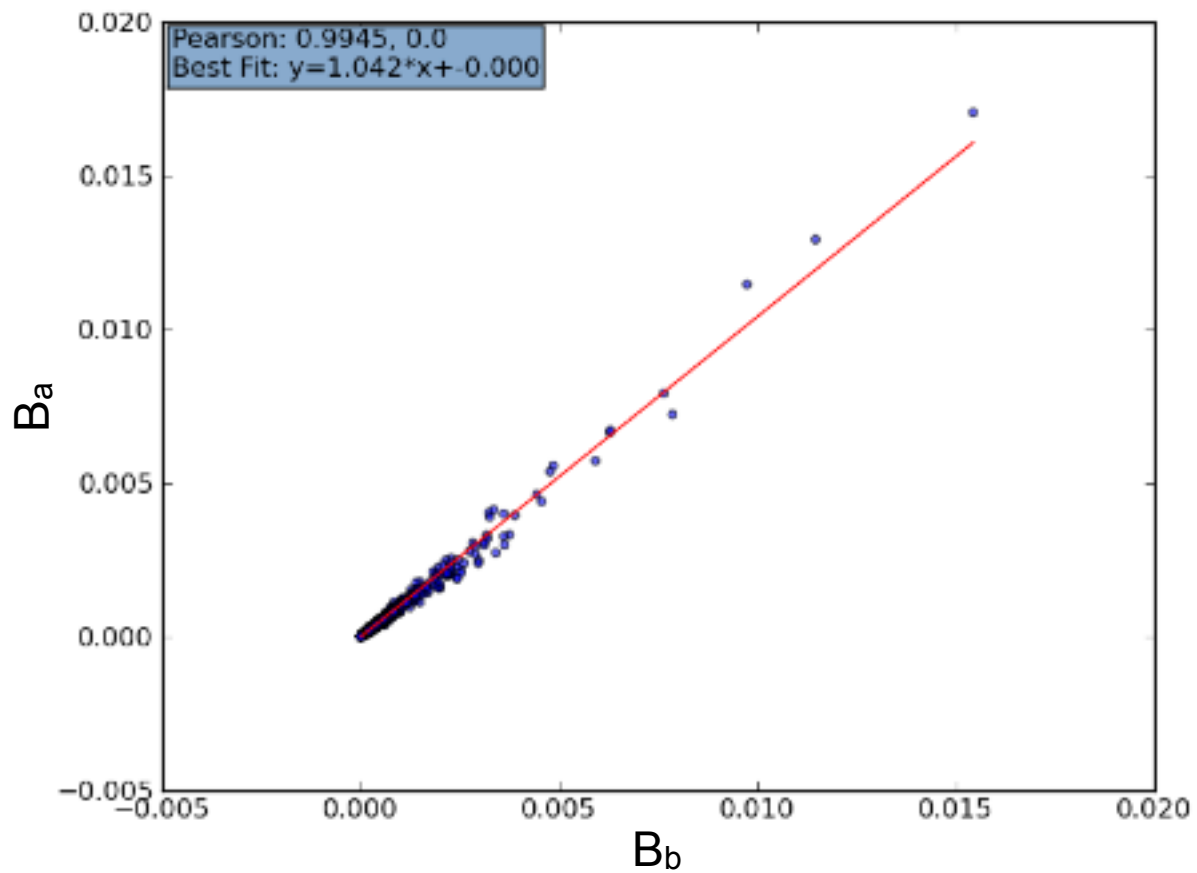**S**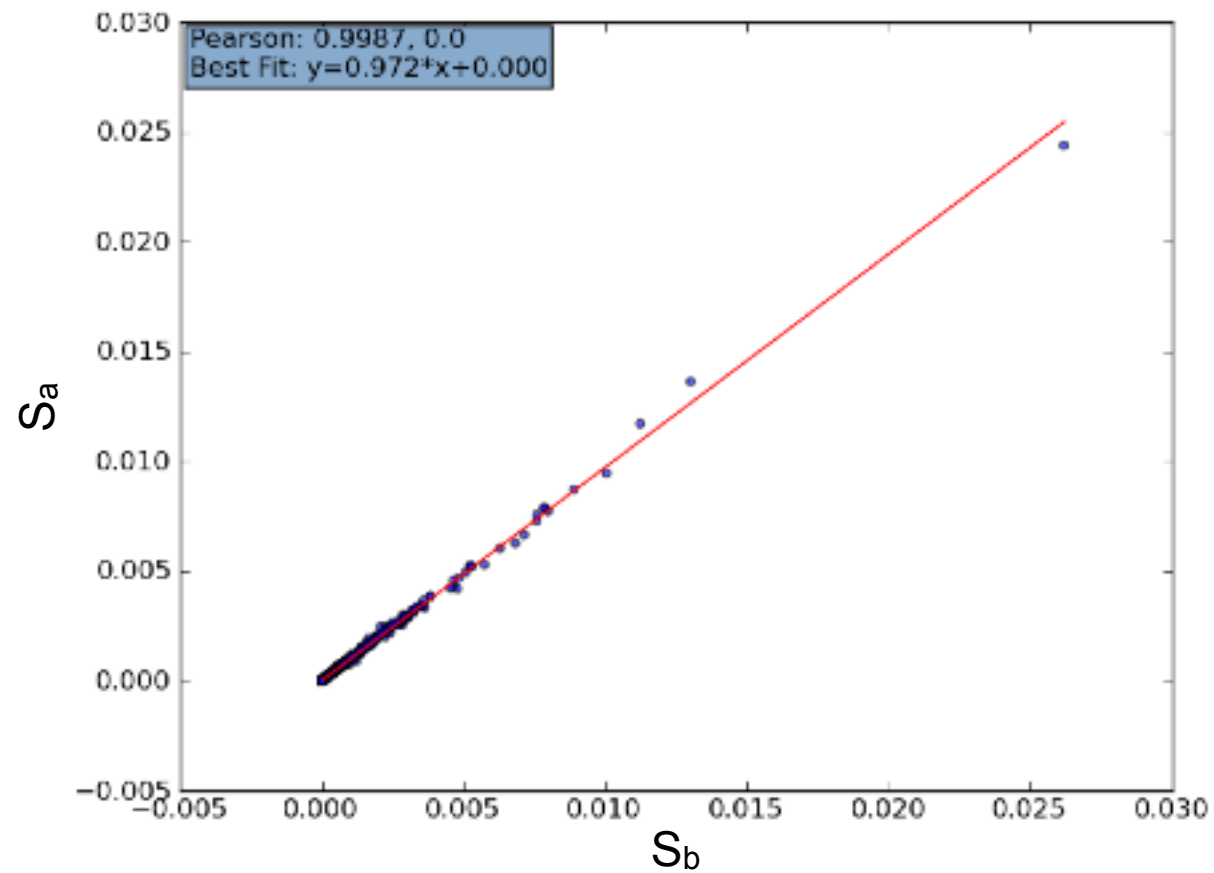

Supplement: Additional file 1 — Comparison of normalized transcript abundance between replicate libraries with respective Pearson correlations. (B) Blood-fed. (S) Sugar-fed. Axes values are in reads transcript-1 library-1. Ba: blood-fed replicate library A. Bb: blood-fed replicate library B. Sa: sugar-fed replicate library A. Sb: sugar-fed replicate library B. The Pearson statistics and equation for the best-fit line are shown in the inset. [file 1471-2164-12-82-S1.PDF]

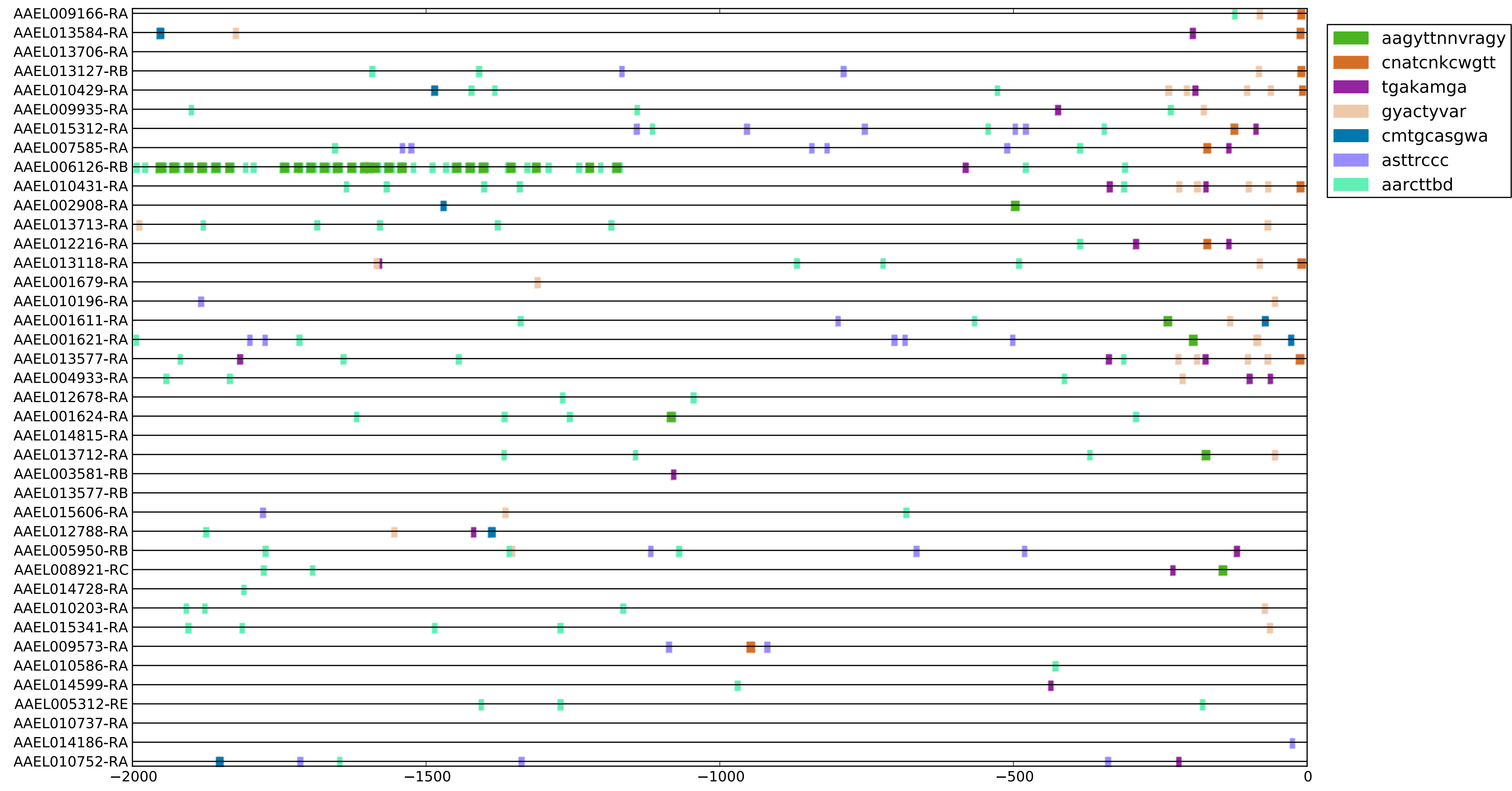

Supplement: Additional File 4 — Motif map of putative CREs discovered by SCOPE using transcripts detected significantly only in blood fed female Ae. aegypti. Locations of representative SCOPE-derived CRE motifs in the 2000 bp upstream of the annotated translational start site in the 40 transcripts detected significantly only in B. Transcript names on the left are ordered from most (top) to least (bottom) abundant. [file 1471-2164-12-82-S4.PDF]
