# Supplementary material for: RNA-seq analyses of blood-induced changes in gene expression in the mosquito vector species, Aedes aegypti
Source: BMC Genomics. 2011 Jan 28;12:82. doi: 10.1186/1471-2164-12-82 (PMC3042412; doi:10.1186/1471-2164-12-82)
Supplement: Additional file 5 — List of primers used for real-time RT-PCR validation of RNA-seq based data. [file 1471-2164-12-82-S5.DOC]

| gene | primers | | qPCR efficiency (std) |
| --- | --- | --- | --- |
|  | forward | reverse |  |
| AY539746 (*rp49*) | 5' ACAAGCTTGCCCCCAACT | 5' CCGTAACCGATGTTTGGC | 95.449 (4.999) |
| AAEL006138 | 5' GCTACAAGTACAACAACCA | 5'TGTGACGTTGTAGACATACT | 96.025 (5.498) |
| AAEL013284 | 5' AAAGTTGAACTCTCTCAGG | 5' GTCCATCATTAGTTGTGTC | 93.300 (5.919) |
| AAEL013707 | 5' GTCAGTTAGTAGCAGTCA | 5' CTCATCCAATTCCAACAG | 97.713 (4.582) |
| AAEL001806 | 5' CGGAGGACTGTCTTCTTTCG | 5' CTTAGGACGGATCCTTGCAC | 98.150 (4.508) |
| AAEL014734 | 5' GAGTCTAACGTCAGTATCAA | 5' GTTACCAAATGAGTTCTACC | 90.525 (1.978) |
| AAEL011470 | 5' ACTATCTGATTGGAGTAACG | 5' GTAGTTCCATTCGCAGTA | 93.938 (4.929) |
| AAEL013005 | 5' ATGTGGACTTTGTACAGG | 5' TCTCCTTCATACTCGTAATC | 95.563 (6.413) |
| AAEL002565 | 5' TACAAGTTCAAGACCGAAGGA | 5' TAGGCGAAGCAGGAGATG | 89.313 (5.384) |
| AAEL008848 | 5' CAACGCCATCCTGAACCT | 5' GACACGACCGACTTGAACT | 96.700 (6.492) |
| AAEL012175 | 5' CATGAACTACACCATCATC | 5' GAGTGGAGATAGAACACATC | 96.550 (6.791) |
| AAEL011871 | 5' GCAGTATTACAATCCCTACT | 5' AAGATGATACCCAAAGATT | 93.675 (5.535) |
| AAEL006425 | 5' ACCCGAACAAGATGAACCAG | 5' TGGTTGTACGATGGGACGTA | 97.600 (4.695) |
| AAEL008701 | 5' CACGAAAACCGGAATGTACC | 5' GCAGCGTACTCTTGTTGTGC | 96.388 (5.977) |

Supplemental Table 3. Primer sets used for real-time RT-PCR.
